# Editorial for the Special Issue “Animal Models of Parkinson’s Disease and Related Disorders”

**DOI:** 10.3390/ijms21124250

**Published:** 2020-06-15

**Authors:** Yuzuru Imai

**Affiliations:** Department of Research for Parkinson’s Disease, Juntendo University Graduate School of Medicine, Tokyo 113-8421, Japan; yzimai@juntendo.ac.jp; Tel.: +81-3-6801-8332

Parkinson’s disease (PD) is the second most common neurodegenerative disorder characterized by age-dependent motor dysfunction and degeneration of the midbrain dopaminergic neurons. The deposition of neuronal inclusion, named Lewy body (LB), in the affected regions is a pathological feature of PD and related disorders such as dementia with LB (DLB). Lewy body formation is thought to begin with α-synuclein aggregation and fibrillation. Experimental studies based on the knowledge obtained by epidemiological and genetic studies continue challenging researchers to make PD risk predictable and surmountable. In this context, the development of experimental models of PD has contributed to the understanding of PD etiology and the development of therapeutics. The current 11 contributions that comprise this Special Issue highlight the PD-associated phenotypes and their evaluation methods and the development of therapeutic strategies using animal models of PD (Figure 1).

The discovery of the mitochondrial toxin 1-methyl-4-phenyl-1,2,3,6-tetrahydropyridine (MPTP) focused the spotlight on the roles of mitochondria in dopaminergic neurons [1]. Mitochondrial toxin 1-methyl-4-phenyl-1,2,3,6-tetrahydropyridine converted to 1-Methyl-4-phenylpyridinium (MPP^+^) by glial monoamine oxidase B is transported to dopaminergic neurons probably through the dopamine transporter and inhibits the mitochondrial respiratory complex I subunits [2,3]. Researchers often employ MPP^+^ and its precursor, MPTP, to make animal or cellular models of PD. Kinoshita et al. [4] found that the administration of MPTP to mice facilitates hippocampal memory extinction, which may reflect cognitive impairment in PD. Using their MPTP model, they reported that serotonin receptor agonists prucalopride and velusetrag could improve the cognitive function by stimulating the cAMP/CREB pathway in the hippocampus [5]. 

Another neurotoxin, 6-hydroxydopamine (6-OHDA), is a dopamine analogue which produces selective damage to dopaminergic neurons by generation of reactive oxygen species (ROS). Unlike MPTP, 6-OHDA does not cross the blood–brain barrier and is used to induce the degeneration of the nigrostriatal pathway by intracerebral stereotactic injection. 6-Hydroxydopamine-induced rodent models are generally unilateral lesion models and exhibit a rotation response by apomorphine. Rosa et al. [6] reported an easy method to evaluate the nigrostriatal degeneration by 6-OHDA using tail suspension behavior without apomorphine challenge. Although aging is a major risk factor for PD, most studies using neurotoxin models do not evaluate aging effects. Barata-Antunes et al. [7] reported that aged rats have a higher susceptibility to 6-OHDA.

The pesticide rotenone is known to inhibit the mitochondrial complex I, generating ROS. Chronic systemic exposure to rotenone has been reported to reproduce selective nigrostriatal dopaminergic degeneration with LB-like α-synuclein-positive inclusions in rat [8]. However, the employment of rotenone rat models is limited due to the fact of their inconsistent results [8]. Miyazaki et al. [9] developed a new rotenone mouse model which exhibited motor deficits and α-synuclein-positive neuronal inclusions in the substantia nigra pars compacta, the dorsal motor nucleus of the vagus, and the intestinal myenteric plexus, reproducing a neuropathological feature of PD.

Hyposmia, constipation, and rapid eye movement sleep behavior disorder are considered prodromal symptoms of PD that often precede motor symptoms. These phenotypes are particularly important in developing disease-modifying therapies that prevent the onset or control progression of PD. Taguchi et al. [10] reviewed the current animal models that would reproduce the prodromal symptoms. Progress of PD is often accompanied by depression. Hao et al. [11] described the characteristics of depression models and their evaluation. 

A synaptic vesicle-binding protein, α-synuclein, is a key protein to produce PD symptoms, forming LBs in the associated neurons. Recent cellular and animal model studies have revealed that α-synuclein has a prion-like property, ascending from peripheral to central neural circuits. Mori et al. [12] reviewed the key studies that examined the roles of phospholipids in terms of α-synuclein aggregation. Missense mutations of β-synuclein, a homologue of α-synuclein, were found in sporadic and familial DLB. Fujita et al. [13] discussed the possible roles of β-synuclein and therapeutic strategies based on their findings using transgenic mice expressing a pathogenic β-synuclein. 

Mutations of *PINK1* and *Parkin* genes cause early-onset familial PD [14,15]. The gene products, PINK1 and Parkin, regulate the quality control of mitochondria through the arrest of mitochondrial transport and autophagic removal of damaged mitochondria [16]. Torii et al. [17] reviewed the roles of a hypoxia-inducible factor (HIF)-1 negative regulator, Inhibitory PAS domain protein (IPAS), in the PINK1 and Parkin pathway using cultured cells and mice.

*Drosophila* is a powerful tool for genetics and has revealed the molecular relationship between PINK1 and Parkin in mitochondria [16]. *Drosophila* is now commonly used as PD models to evaluate genetic association. Elvira et al. [18] reported the suppression of general protein synthesis by eIF2α phosphorylation though protein kinase RNA-like endoplasmic reticulum kinase (PERK) activation is protective against dopaminergic neuron loss in *Drosophila*. Historical perspective of overall PD models was also well summarized by Chia et al [19].

In summary, all articles appearing in this Special Issue cover the interesting and current topics in PD model studies. Although most PD models do not faithfully reproduce all aspects of this disease, PD model studies would advance our knowledge and promote the development of drugs and therapeutic strategies, receiving new inputs from clinical studies. This Guest Editor would like to thank all of the authors for their contributions to this Special Issue and expects significant advancement to our knowledge of PD in future studies.

## Figures and Tables

**Figure 1:**
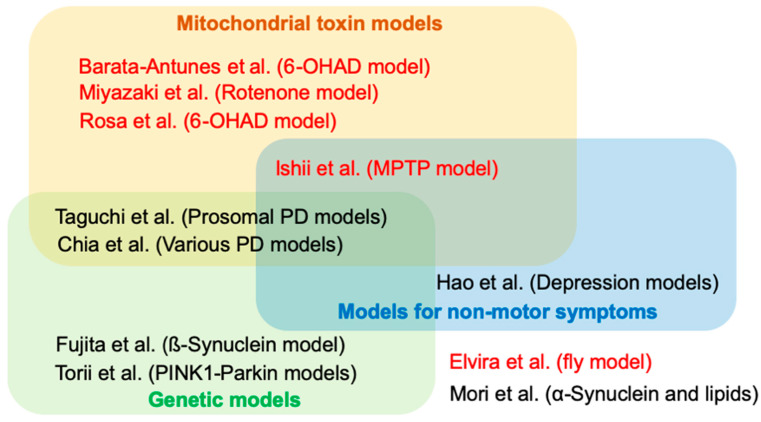
Summary of papers in this Special Issue. Articles in red and in black indicate original and review articles, respectively. 6-OHDA = 6-hydroxydopamine, MPTP = 1-methyl-4-phenyl-1,2,3,6-tetrahydropyridine, PD = Parkinson’s disease.

## Abbreviations

6-OHDA	6-Hydroxydopamine
DLB	Dementia with Lewy bodies
HIF-1	hypoxia-inducible factor-1
IPAS	Inhibitory PAS domain protein
LB	Lewy body
MPP+	1-Methyl-4-phenylpyridinium
MPTP	1-Methyl-4-phenyl-1,2,3,6-tetrahydropyridine
PD	Parkinson’s disease
PERK	Protein kinase RNA-like endoplasmic reticulum kinase
ROS	Reactive oxygen species
